# Three-unit posterior monolithic fixed dental prostheses made from high-translucent shade-graded zirconia: 3-Year results from a prospective clinical pilot study

**DOI:** 10.1007/s00784-024-06084-5

**Published:** 2024-12-16

**Authors:** Jan-Frederik Güth, Christine Keul, Anja Liebermann, Josef Schweiger, Daniel Edelhoff, Oliver Schubert

**Affiliations:** 1 Department of Prosthetic Dentistry at the Center for Dentistry and Oral Medicine (Carolinum), Goethe University Frankfurt am Main, D-60596 Frankfurt am Main, Germany; 2 Department of Prosthetic Dentistry, University Hospital, LMU Munich, Goethestraße 70, D-80336 Munich, Germany; 3 Department of Prosthetic Dentistry, University Hospital of Cologne, D-50931 Cologne, Germany

**Keywords:** 5Y-PSZ, All-ceramic, CAD/CAM, High-translucent zirconia, FDP, Fixed dental prostheses, Monolithic ceramic

## Abstract

**Objectives:**

To assess the clinical performance of tooth-supported 3-unit fixed dental prostheses (FDPs) made from shade-graded monolithic 5Y-PSZ (partly stabilized zirconia) zirconia in terms of survival rate and the quality of restorations based on modified FDI criteria over three-years.

**Materials and methods:**

High-translucent shade-graded monolithic zirconia (Lava Esthetic, Solventum Dental Solutions) was used to manufacture maxillary or mandibular three-unit FDPs in the posterior region (*N* = 22) employing subtractive milling system (Amann Girrbach). All FDPs were bonded with a universal resin cement (Rely X Universal, Solventum Dental Solutions) and evaluated 4 weeks after cementation (baseline) and after 1, 2, and 3 years. The primary objective was to assess the survival and complication rates of the restorations. Furthermore, the quality of the restorations was evaluated based on selected and modified FDI (World Dental Federation) criteria, which encompass functional, aesthetic, and biological parameters. FDI criteria were analyzed using the Fisher-Freeman-Halton exact test.

**Results:**

Twenty-one patients were examined at 1 year, 2 years, and 3 years. The survival rate was 100%. No mechanical complications were observed. A total of 3 biological complications occurred. These were successfully managed without any residual functional impairment. All FDI criteria were found to be clinically acceptable or better.

**Conclusions:**

Monolithic shade-graded zirconia FDPs demonstrated 100% survival at 3 years with a low complication rate.

**Clinical relevance:**

Fixed 3-unit FDPs made of high-translucent monolithic zirconia might be a viable treatment option in the posterior region, preventing the chipping phenomenon and providing favorable aesthetics while allowing for an efficient digital workflow.

## Introduction

The digital transformation of dentistry, driven by advances in computer-aided design and manufacturing (CAD/CAM), has fundamentally reshaped traditional workflows [1]. Producing dental restorations from prefabricated materials using standardized processes reduces human error, improves precision, facilitates customization, and accelerates processing times. Integrating CAD/CAM systems into dental practices is fostering a streamlined, patient-centered approach [2].

Due to their material characteristics and esthetic potential, all-ceramic materials have become a remarkable success in dentistry with a wide range of applications in modern dentistry - and are increasingly replacing the previous gold standard, metal ceramics [3]. In addition to glass-ceramic-based materials, different generations of zirconia-based ceramics are available on the dental market. Zirconia, with its outstanding physical properties, and excellent biocompatibility, meets many of the requirements of modern dentistry and dental technology [4]. It is employed as a framework and monolithic material in single crowns and fixed dental prostheses, in removable prosthetics, and in the fabrication of dental implants [5].

Nevertheless, there are some limitations regarding zirconia restorations. Conventionally veneering zirconia is labor-intensive, and the outcome is highly dependent on the individual skills of the dental technician. The restorations are prone to veneering fracture, clinically referred to as “chipping” [6]. Consequently, there is a persistent trend in the dental market towards monolithic zirconia materials. Advantages include, inter alia, a streamlined and efficient production process, enhanced precision, and the reduced risk of chipping [7].

In turn, another problem emerged. Conventional zirconia contains 3 mol% yttria, which enables the stabilization of the tetragonal phase at room temperature. The 3 mol% yttria-stabilized tetragonal zirconia polycrystal (3Y-TZP) can form a transformation zone that provides protection against the formation of cracks. This transformation toughening contributes to a high fracture resistance [8]. However, the primary disadvantage of the original 3Y-TZP is its opacity [9]. Therefore, despite the increasing use of monolithic zirconia restorations, especially in the posterior region, their use has remained limited due to these aesthetic considerations [7].

To overcome these drawbacks, another iteration of zirconia has been developed with an increased yttria content. The material is fabricated with 5 mol% yttria, which partially stabilizes the cubic phase (about 50%). This zirconia (5Y-PSZ) has been designated “cubic zirconia” or “translucent zirconia” due to its enhanced optical characteristics [9]. The introduction of 5Y-PSZ provides promising characteristics, with flexural strength and translucency between those of lithium disilicate and 3Y-TZP zirconia [10]. However, there is almost no evidence on the clinical performance of restorations out of this category of zirconia.

Therefore, the aim of the present prospective, clinical study was to test the clinical outcomes of 3-unit posterior FDPs made from high-translucent monolithic 5Y-PSZ with shade-grade technology and inherent fluorescence. The hypothesis was that FDPs have high survival rates of up to 3 years with a low complication rate and high patient satisfaction.

## Materials and methods

### Study design and patient selection

This study was designed as a pilot single arm trial to address the scarcity of in vivo scientific information on the tested shade-graded monolithic zirconia ceramic. It was approved by the ethics committee responsible (UE No 19–0243). The study was conducted in the Department of Prosthetic Dentistry at the University Hospital of LMU Munich. Patients requiring a three-unit fixed partial denture (FPD) in the premolar and molar region were recruited. The assessment of the presence or absence of craniomandibular dysfunctions was conducted as proposed by Ahlers and Jakstat [11].

Inclusion criteria were met when the patient was requiring a three-unit posterior bridge, had healthy/treated periodontal status (maximum tooth mobility: 1), was aged 18–99 years, agreed to participate in the study and signed an informed consent form.

Patients with known allergies to any product used during the study, untreated periodontal disease, or who were not available for the entire duration of the study, who were participating in another dental clinical trial, or who were pregnant or breastfeeding were excluded.

### Clinical treatment

**Fig. 1:**
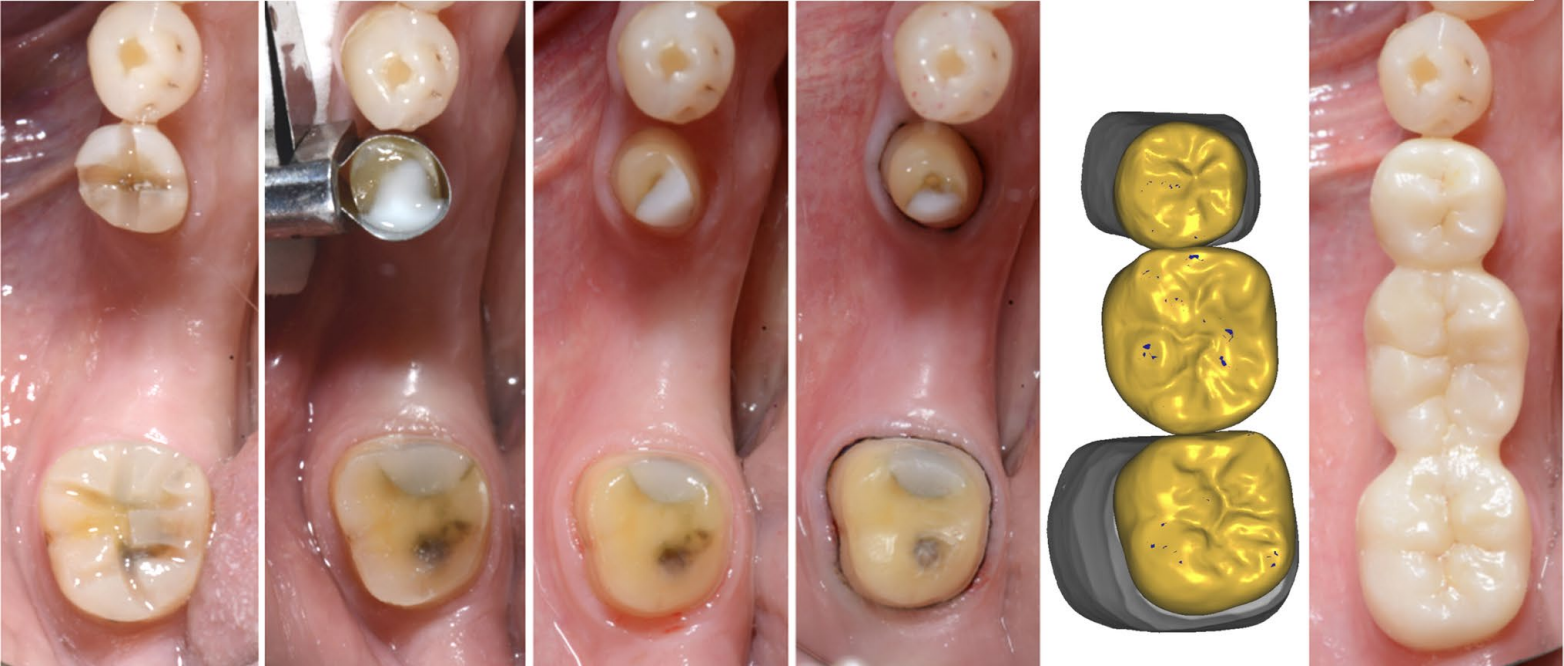
The clinical workflow (example). Preparation of occlusal grooves, renewal of a composite filling, final preparation, cording, CAD of the FDP, and final restoration (from left to right)

Twenty-five pre-selected patients were examined but only 22 patients, 16 males (73%) and 6 females (27%), met the inclusion criteria, provided informed consent, and were finally included. A total of 22 posterior three-unit fixed partial dentures (FDPs) were fabricated, comprising 44 abutment teeth. Of these, 18 (41%) were located in the maxilla and 26 (59%) in the mandible. Four FDPs comprised the first premolar and first molar, 18 were attached to the second premolar and molar respectively. The treatment was performed by skilled and calibrated clinicians (JG, OS). The clinical workflow is illustrated in Fig. 1. The abutment teeth were prepared according to the manufacturer’s recommendations for the respective restorative material. The requirements were to ensure a wall thickness of 0.8 mm, and the connector diameter had to be greater than 14 mm^2^. Restorations were delivered between July 2019 and June 2020. Impressions were taken with a fast-setting polyether impression material (Impregum Penta Super Quick, Solventum Dental Solutions, Seefeld, Germany). Bite registration was performed using a scannable registration silicone material (Imprint 4 Bite, Solventum Dental Solutions). Temporary restorations were manufactured chairside (ProTemp, Solventum Dental Solutions) and cemented with a eugenol-free temporary cement (RelyX Temp NE, Solventum Dental Solutions).

### Fabrication of the fixed dental prostheses

Plaster casts were manufactured (Resin Rock, Whipmix, Louisville, KY, US) and were with a laboratory scanner (S900, Zirkonzahn, Gais, Italy). All FDPs were CAD designed and fabricated by skilled and calibrated dental technicians (JS, CS).

The monolithic FDPs were virtually designed using CAD/CAM software (Modelier v.6173_6959_x64, Zirkonzahn) and milled from LAVA Esthetic partially sintered blanks (Solventum Dental Solutions) using a five-axis milling unit (Ceramill Motion 2, Amann Girrbach, Koblach, Austria). LAVA Esthetic is a 5 mol% yttria-stabilized cubic zirconia material presenting a micro-hardness of 1,200 HV [Vickers hardness], an elastic modulus of 216 GPa, and a flexural strength of 800 MPa. It is characterized as high-translucent and features inherent fluorescence. Milled restorations were sintered in a furnace (LHT 02/16; Nabertherm, Lilienthal, Germany), applying manufacturer’s recommendations. The restorations were finalized in terms of stain and glaze firing.

### Delivery of the fixed dental prostheses

Prior to cementation, fit (light body silicone; Fit&Test Voco, Cuxhaven, Germany) and occlusal and interproximal contacts of the FDPs tested. If necessary, the occlusion was adjusted, and the surfaces were polished carefully with ceramic polishers or glaze-fired again. The FDPs were cleaned with ethanol, and the abutment teeth were cleaned with a universal prophy paste (Prophy Paste Cleanic without fluoride, Kerr Dental, Brea, CA, USA). Air-abrasion of the intaglio surfaces was performed with 50 μm abrasive alumina particles at 1 bars (0.1 MPa) [12]. No further chemical conditioning was conducted. All FDPs were cemented employing self-conditioning, dual-curing universal resin cement (RelyX Universal, Solventum Dental Solutions). Excess cement was removed, and occlusion was re-assessed. Following the treatment, but for a limited time span, 23% of the teeth exhibited mild hypersensitivity, while 11% displayed moderate hypersensitivity. After delivery, an operator’s questionnaire on the handling of the adhesive resin cement was completed on “satisfaction with the cementation“, “satisfaction with excess removal”, “ease of mounting of the mixing tip”, and “extrusion force of the mixing tip”, using a Likert type scale.

### Clinical examinations

At the baseline examination, conducted four weeks post-delivery, and at the subsequent follow-up examinations at one year, two years, and three years post-delivery, the FDPs were clinically examined. An overview of evaluations during the treatment and questionnaires are given in Table 1.

**Table 1:** An overview of evaluations during the treatment and questionnaires

Evaluation	Point in time
Fit of restoration	Prior to restoration placement
Craniomandibular dysfunctions (Ahlers and Jakstat 2007)	Restoration placement appointment
Restoration evaluation per FDI criteria	Baseline, 1-, 2-, and 3-year follow-ups
Post-operative sensitivity	Baseline
Pocket depth (6-point measurement)	Prior to restoration placement, and at 1-, 2-, and 3-year recalls
Radiographs	At 3-year follow-up
Operator Questionnaire	After restoration placement
Patient Questionnaire	At 1-year follow-up

The esthetic, functional, and biological outcomes were evaluated in accordance with the modified FDI criteria and periodontal probing depth (PPD) [13, 14]. The modified FDI-criteria (World Dental Federation) are displayed in Table 2. Marginal adaptation was tested using an explorer probe (Fissures explorer 150EX, Deppeler, Rolle, Switzerland) [14].

**Table 2:** Modified FDI criteria

FDI Criteria	Property	Score
Color Stability	Esthetic Property	1 Very Good
		2 Good
		3 Acceptable
		4 Unacceptable
Fracture	Functional Property	1 No
		2 Yes
Retention loss	Functional Property	1 No
		2 Yes
Chipping	Functional Property	1 No
		2 Yes
Wear	Functional Property	1 No
		2 Attrition/Facets
		3 Perforation of the Restoration
Periodontal response	Biological Property	1 No plaque and no inflammation
		2 Plaque and no gingivitis
		3 Plaque and gingivitis
Recurrence of caries	Biological Property	1 No
		2 Yes
Marginal adaptation	Biological Property	1 No detectable gap
		2 Marginal gap < 150 μm
		3 Marginal gap < 250 μm
		4 Marginal gap > 250 μm
Marginal Discoloration	Esthetic Property	1 No
		2 Yes, but removable
		3 Yes, but not removable
Vitality	Biological Property	1 Vital
		2 Non vital
		1 Percussion negative
		2 Percussion positive
Postoperative (hyper-) sensitivity(at baseline)	Biological Property	1 No
		2 Yes

Prior to these examinations, a calibration was performed to instruct and train the examining dentists. To reduce potential bias, the FDPs were examined by two independent clinicians (AJ, CK) who were not involved in the reconstructive treatment. Survival of the FDPs was classified as “FDP in situ and in full function” at the follow-up visits. Radiographs were taken at the 3-year follow-up.

All data were subjected to detailed analysis. At the one-year follow-up, the patients were queried regarding their satisfaction with the functionality and the esthetic outcome using a 5-point Likert type scale. The questionnaire evaluated general satisfaction, esthetic satisfaction, comfort, chewing comfort, and satisfaction with abilities to speak, also using a scale from 1 (very good) to 5 (very bad).

### Statistical analysis

Baseline parameters were analyzed using mean, standard deviation, median, minimum and maximum. The change in parameters from baseline was evaluated using descriptive statistics. The primary outcome of this trial was the evaluation of the overall survival of restorations. Complications were given in percentage. Modified FDI criteria scores were analyzed using the Fisher-Freeman-Halton exact test. For statistical evaluation, the software SPSS (Statistics 24.0, SPSS Inc., Stanford, USA) was used and level of significance was set at *p* < 0.05.

## Results

**Fig. 2:**
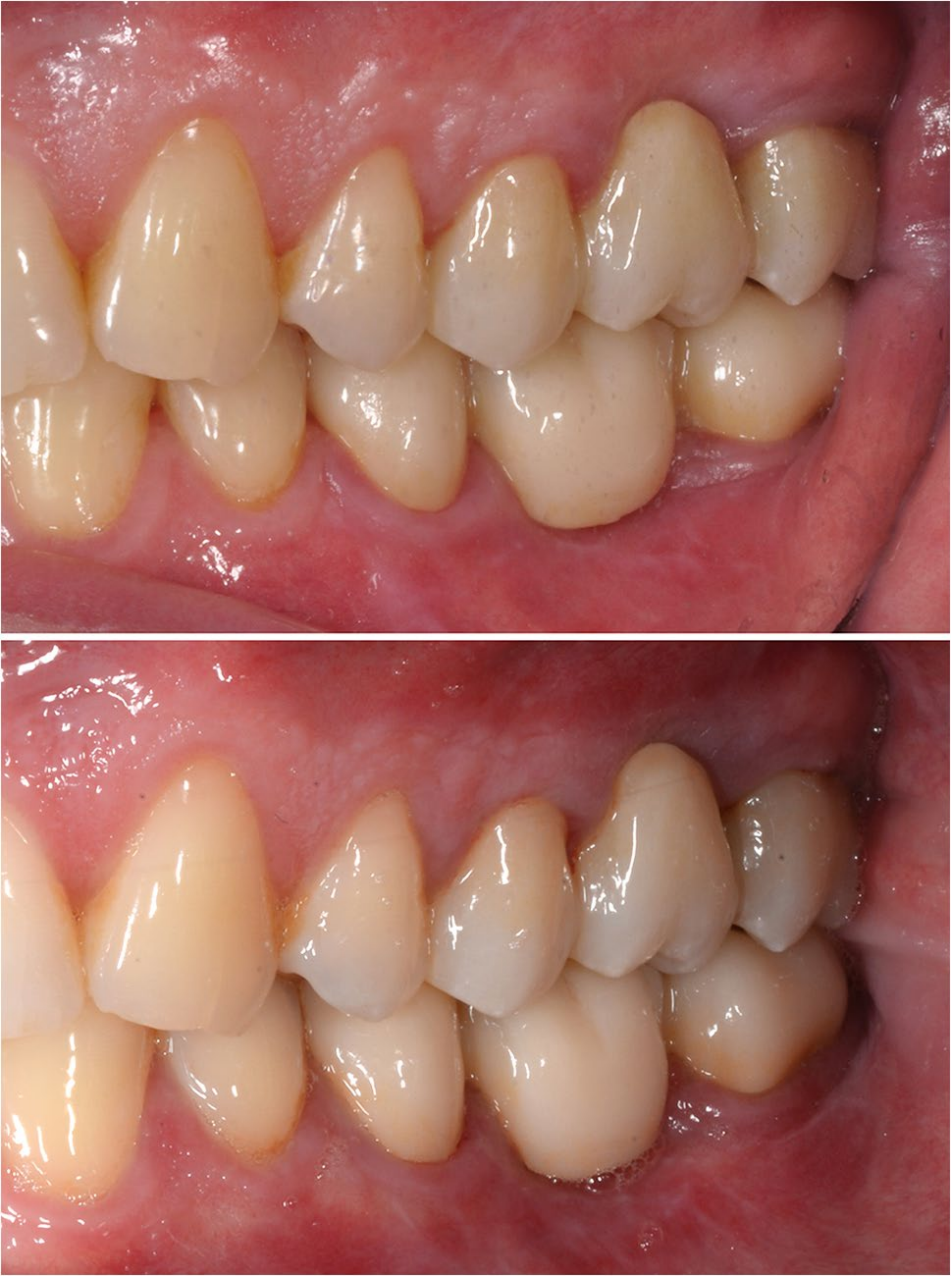
FDP in the left upper jaw comprising the second premolar to the second molar at baseline (top) and at 3 years (bottom)

21 FDPs, comprising 42 abutment teeth, were examined up to after three years. Of these, 9 (43%) were in the maxilla and 12 (57%) in the mandible. 17 patients were evaluated in the defined interval (36 months ± 31 days), 4 patients exceeded the recall window due to various issues. Only one patient did not take part in the 3-year follow-up, as he had relocated to a distant location. The results were distinguished in FDP and abutment teeth related aspects are summarized in Tables 3 and 4. Figure 2 shows an example of an FDP over time.

**Table 3:** Results with respect to FDPs (*n* = 22)

FDI Criteria	Examination time point		Clinical Performance Score					Significancy
			1	2	3	4	5	
Survival rate	BL	n	22					1
		%	100%					
	36 Months	n	21					
		%	100%					
Color Stability	BL	n	6	16				0.43
		%	27.3%	72.7%				
	36 Months	n	6	14	1			
		%	28.6%	66.7%	4.8%			
Fracture	BL	n	22					1
		%	100%					
	36 Months	n	21					
		%	100%					
Retention loss	BL	n	22					1
		%	100.0%					
	36 Months	n	21					
		%	100%					
Chipping	BL	n	22					1
		%	100%					
	36 Months	n	21					
		%	100%					
Wear	BL	n	22					< 0.001*
		%	100%					
	36 Months	n	13	8				
		%	61.9%	38.1%

**Table 4:** Results with respect to abutment teeth (*n* = 44)

FDI Criteria	Examination time point		Clinical Performance Score					Significancy
			1	2	3	4	5	
Periodontal response	BL	n	34	9	1			0.5
		%	77.3%	20.5%	2.3%			
	36 Months	n	33	6	3			
		%	78.6%	14.3%	7.1%			
Recurrence of caries	BL	n	44					0.49
		%	100.0%					
	36 Months	n	41	1				
		%	97.6%	2.4%				
Marginal adaptation	BL	n	30	14				< 0.001*
		%	68.2%	31.8%				
	36 Months	n	12	30				
		%	28.6%	71.4%				
Marginal Discoloration	BL	n	42	2				1
		%	95.5%	4.5%				
	36 Months	n	41	1				
		%	97.6%	2.4%				
Vitality	BL	n	40	4				0.736
		%	90.9%	9.1%				
	36 Months	n	37	5				
		%	88.1%	11.9%				
Percussion	BL	n	44					1
		%	100.0%					
	36 Months	n	42	0				
		%	100.0%					

No chipping, loss of retention, or fractures occurred, resulting in zero mechanical complications and a 100% survival rate. Three adverse events, i.e., biological complications, all rated “non-serious”, occurred during the 3 years of observation, representing a total prevalence of 14.3% and an annual rate of approximately 4.76%. In one patient, an endodontic treatment of one abutment tooth was conducted. Another adverse event, classified “sensitivity due to occlusal trauma”, was successfully treated by minor occlusal adjustments. Caries was found in one FDP abutment tooth, necessitating filling therapy (*p* = 0.49). After treatments, all patients were without symptoms and the FDPs could remain in full functional service.

The parameters “marginal discoloration” (*p* = 1) and “percussion” (*p* = 1) did not change. Compared to baseline, clinical parameters “periodontal response” (*p* = 0.5), “color stability” (*p* = 0.43), “recurrence of caries” (*p* = 0.49), and “tooth vitality” (*p* = 0.736) altered, but not significantly. Visible signs of “wear” occurred in 16 restorations on anchor teeth (*p* < 0.001), and “marginal adaptation” was perceived less favorable (*p* < 0.001).

The probing depth (6-point measurement) exhibited a subtle variation over time, with mean values ranging from 2.2 (± 1.0) mm to 2.6 (± 1.0) mm at the one-year mark and from 2.3 (± 0.7) mm to 2.8 (± 1.1) mm after three years, contingent on the probing site.

The operator questionnaire on the operability of the resin cement showed the highest levels of satisfaction in all aspects. The patients´ questionnaire, which was handed out one year after the intervention, revealed that patients had a generally positive assessment of the FDP, with results ranging from 1.0 (ability to speak) to 1.6 (chewing comfort) on average. Two patients expressed discontent with the chewing comfort and one felt general discomfort.

## Discussion

The FDPs had a 100% survival rate after three years, with no mechanical complications, indicating notable clinical success. The positive results support the study’s hypothesis, and the complete absence of chipping, likely due to the monolithic design, can be considered a key factor for prognosis and longevity.

Regarding the modified FDI parameters, all restorations were rated satisfactory or better at the 3-year follow-up evaluation. Minor deviations from the excellent score for some aspects were observed from baseline to the 3-year follow-up. This is in line with previous studies [15–17]. Periodontal response was within the expected range.

The decrease in vitality resulted from a tooth needing root canal treatment, likely due to preparation trauma and pulp irritation. The treatment was successful, and the restoration remained fully functional. FDP abutment teeth are more prone to endodontic complications due to invasive preparation and associated trauma, sometimes unavoidable due to axial divergences. This is not considered specific to this study [18]. One patient suffered sensitivity due to occlusal trauma, which is not uncommon after the delivery of a restoration, especially in terminal three unit FDPs [19], but could be effectively treated. A carious lesion was found on the buccal cervical aspect of the second abutment tooth in one patient. The root caries was treated with glass ionomer cement, and the patient remained asymptomatic afterward.

The evaluator’s assessment of the color stability of the restorations was similar to the assessment at baseline. The observation that marginal adaptation was perceived to be less favorable than at baseline must be qualified by two important factors. First, there were changes from “not detectable” to “<150 µm”, i.e., just perceptible, which represents minimal change [14]. The number of calibrated examiners was reduced from two to one due to an employer change, which may introduce some variability in measurements. To minimize further bias, the study team chose not to add a new second evaluator. Future recalls may help assess these effects more accurately.

Visible signs of wear could be detected in 10 restorations, most likely due to abrasion of the glaze layer and within the time frame of two years of clinical use. This is therefore considered an expected event [20].

The operator questionnaire on the handling of the resin cement showed excellent satisfaction; the questionnaire given to the patients showed that they had a generally positive opinion of the FDP - both of which can be considered a great success.

Veneered zirconia FDPs have proven to be reliable under clinical conditions, with high long-term success rates, but have the disadvantages mentioned above, such as chipping and laborious fabrication [6, 7, 16, 17, 21]. Nevertheless, all new materials or material combinations to date have to compete with metal-ceramic FDPs, which are the most proven and considered the gold standard for posterior restorations [3]. The survival rates of these FDPs are notable, with 93.8% at five years and 89.2% at ten years of follow-up.

Long-term data on monolithic zirconia FDPs remain limited. Koenig et al. reported a 100% survival rate for second-generation zirconia monolithic posterior restorations on 95 teeth and implants, though all 13 FDPs were implant-supported, making direct comparisons cautious [22]. In a clinical trial, Habibi et al. found monolithic restorations had slightly better performance, with a 96.7% survival rate and 93.8% success rate after three years [23]. Pontevedra et al. recently reported survival rates at three years for 30 three-unit posterior fixed partial dentures (FPDs) made from 3Y-TZP monolithic zirconia of 90%. They concluded that monolithic zirconia in combination with a complete digital workflow could represent a viable alternative in the posterior regions [15].

Mid- to long-term clinical data on cubic zirconia FDPs are, to the authors’ knowledge, still unavailable. However, this trial’s positive results align with existing research. An in vitro study by Hensel et al. showed that 3-unit FDPs made from this material withstood high fracture loads of up to 1,541.9 (± 645.8) N before and 1,705.8 (± 248.1) N after artificial aging, respectively [24], which may indicate that even after longer periods of clinical service, high survival rates can be expected. Park et al. found that low-temperature degradation (LTD) did not affect Lava Esthetic zirconia’s material properties. They also observed that the hardness increased from the incisal to the core layer, while brightness decreased [25]. Additionally, Lava Esthetic caused less antagonist wear than lithium silicate and lithium disilicate ceramics [26].

The connector has been designed according to the manufacturer’s recommendations, which may be important since the design of 3-unit fixed dentures, particularly the connector height, may affect fracture strength also in this 5Y-PSZ , according to Kim et al. [27]. The benefit of monolithic zirconia FDPs in this respect is the absence of veneering, which allows a larger connector diameter, particularly in the vertical dimension. As a result, the full diameter of the connector contributes to mechanical strength.

Although this is a very relevant research topic, the present study has limitations in terms of the number of cases and observation period. One dropout occurred and the coronavirus pandemic had an impact on the time windows for the follow-up as well. The significance of the results must be considered against this background. Nonetheless, these findings remain important. To the authors’ knowledge, this is the first clinical data on the three-year performance of high-translucent, cubic zirconia in 3-unit FDPs, showing promising results. Even counting dropouts as failures, the three-year survival rate would be 95.5%.

## Conclusion

The findings of this clinical study suggest that CAD/CAM-manufactured 3-unit zirconia fixed dental prostheses made from a high-translucent shade-graded monolithic zirconia (Lava Esthetic) may represent a viable treatment option for the posterior region over a minimum of three years. All FDPs survived without mechanical complications. The FDI criteria examined were rated as clinically acceptable or better. However, to provide clinicians with the certainty and security they require in the use of the advantages of this and similar types of translucent zirconia for multi-unit fixed dental prostheses, further clinical data on performance and survival rates need to be collected with larger samples and over longer periods of time.

## Data Availability

No datasets were generated or analysed during the current study.

## References

[1] Alghazzawi TF (2016) Advancements in CAD/CAM technology: options for practical implementation. J Prosthodontic Res 60(2):72–84. 10.1016/j.jpor.2016.01.003 10.1016/j.jpor.2016.01.003 26935333

[2] Beuer F, Schweiger J, Edelhoff D (2008) Digital dentistry: an overview of recent developments for CAD/CAM-generated restorations. Br Dent J 204(9):505–511. 10.1038/sj.bdj.2008.350 18469768 10.1038/sj.bdj.2008.350

[3] Heintze SD, Rousson V (2010) Survival of zirconia- and metal-supported fixed dental prostheses: a systematic review. Int J Prosthodont 23(6):493–502 21209982

[4] Schubert O, Gaissmaier M, Graf T, Schweiger J, Guth JF (2022) Digital veneering techniques for zirconia implant-supported single crowns: bond strength and clinical application. Int J Prosthodont 35(4):545–552. 10.11607/ijp.7563 36125877 10.11607/ijp.7563

[5] Miyazaki T, Nakamura T, Matsumura H, Ban S, Kobayashi T (2013) Current status of zirconia restoration. J Prosthodontic Res 57(4):236–261. 10.1016/j.jpor.2013.09.001 10.1016/j.jpor.2013.09.001 24140561

[6] Pjetursson BE, Sailer I, Makarov NA, Zwahlen M, Thoma DS (2015) All-ceramic or metal-ceramic tooth-supported fixed dental prostheses (FDPs)? A systematic review of the survival and complication rates. Part II: multiple-unit FDPs. Dent Mater 31(6):624–639. 10.1016/j.dental.2015.02.013 25935732 10.1016/j.dental.2015.02.013

[7] Silva LHD, Lima E, Miranda RBP, Favero SS, Lohbauer U, Cesar PF (2017) Dental ceramics: a review of new materials and processing methods. Brazilian Oral Res 31(suppl 1):e58. 10.1590/1807-3107BOR-2017.vol31.0058 10.1590/1807-3107BOR-2017.vol31.0058 28902238

[8] Stawarczyk B, Keul C, Eichberger M, Figge D, Edelhoff D, Lümkemann NJQ (2017) Three generations of zirconia: from veneered to monolithic. Part I. Quintessence Int, 48(5). 10.3290/j.qi.a38057 10.3290/j.qi.a38057 28396886

[9] Zhang Y (2014) Making yttria-stabilized tetragonal zirconia translucent. Dent Mater 30(10):1195–1203. 10.1016/j.dental.2014.08.375 25193781 10.1016/j.dental.2014.08.375 PMC4167579

[10] Kwon SJ, Lawson NC, McLaren EE, Nejat AH, Burgess JO (2018) Comparison of the mechanical properties of translucent zirconia and lithium disilicate. J Prosthet Dent 120(1):132–137. 10.1016/j.prosdent.2017.08.004 29310875 10.1016/j.prosdent.2017.08.004

[11] Ahlers M, Jakstat H (2009) Clinical functional analysis as the first step of a diagnostic cascade: computer-aided individualized assessment, treatment planning, and patient information. J Craniomandib Function 1:57–76

[12] Zhang X, Liang W, Jiang F, Wang Z, Zhao J, Zhou C, Wu J (2021) Effects of air-abrasion pressure on mechanical and bonding properties of translucent zirconia. Clin Oral Invest 25(4):1979–1988. 10.1007/s00784-020-03506-y 10.1007/s00784-020-03506-y 32779015

[13] Hickel R, Peschke A, Tyas M, Mjör I, Bayne S, Peters M, Hiller KA, Randall R, Vanherle G, Heintze SD (2010) FDI World Dental Federation: clinical criteria for the evaluation of direct and indirect restorations—update and clinical examples. Clin Oral Invest 14(4):349–366. 10.1007/s00784-010-0432-8 10.1007/s00784-010-0432-8 20628774

[14] Hickel R, Roulet JF, Bayne S, Heintze SD, Mjör IA, Peters M, Rousson V, Randall R, Schmalz G, Tyas M, Vanherle G (2007) Recommendations for conducting controlled clinical studies of dental restorative materials. J Adhes Dent 9(suppl 1):121–147 17992913 10.1111/j.1875-595x.2007.tb00136.x

[15] Pontevedra P, Lopez-Suarez C, Rodriguez V, Pelaez J, Suarez MJ (2022) Randomized clinical trial comparing monolithic and veneered zirconia three-unit posterior fixed partial dentures in a complete digital flow: three-year follow-up. Clin Oral Invest 26(6):4327–4335. 10.1007/s00784-022-04396-y 10.1007/s00784-022-04396-y PMC9203772 35142924

[16] Suarez MJ, Perez C, Pelaez J, Lopez-Suarez C, Gonzalo E (2019) A randomized clinical trial comparing zirconia and metal-ceramic three-unit posterior fixed partial dentures: a 5-year follow-up. J Prosthodont 28(7):750–756. 10.1111/jopr.12952 30039897 10.1111/jopr.12952

[17] Sailer I, Balmer M, Hüsler J, Hämmerle CHF, Känel S, Thoma DS (2018) 10-year randomized trial (RCT) of zirconia-ceramic and metal-ceramic fixed dental prostheses. J Dent 76:32–39. 10.1016/j.jdent.2018.05.015 29807060 10.1016/j.jdent.2018.05.015

[18] Cheung GS, Lai SC, Ng RP (2005) Fate of vital pulps beneath a metal-ceramic crown or a bridge retainer. Int Endod J 38(8):521–530. 10.1111/j.1365-2591.2005.00982.x 16011770 10.1111/j.1365-2591.2005.00982.x

[19] Parker MW (1993) The significance of occlusion in restorative dentistry. Dental Clin N Am 37(3):341–351 8348990

[20] Stawarczyk B, Özcan M, Schmutz F, Trottmann A, Roos M, Hämmerle CH (2013) Two-body wear of monolithic, veneered, and glazed zirconia and their corresponding enamel antagonists. Acta Odontol Scand 71(1):102–112. 10.3109/00016357.2011.654248 22364372 10.3109/00016357.2011.654248

[21] Forrer FA, Schnider N, Brägger U, Yilmaz B, Hicklin SP (2020) Clinical performance and patient satisfaction obtained with tooth-supported ceramic crowns and fixed partial dentures. J Prosthet Dent 124(4):446–453. 10.1016/j.prosdent.2019.08.012 31902533 10.1016/j.prosdent.2019.08.012

[22] Koenig V, Wulfman C, Bekaert S, Dupont N, Le Goff S, Eldafrawy M, Vanheusden A, Mainjot A (2019) Clinical behavior of second-generation zirconia monolithic posterior restorations: two-year results of a prospective study with ex vivo analyses including patients with clinical signs of bruxism. J Dent 91:103229. 10.1016/j.jdent.2019.103229 31722238 10.1016/j.jdent.2019.103229

[23] Habibi Y, Dawid MT, Waldecker M, Rammelsberg P, Bömicke W (2020) Three-year clinical performance of monolithic and partially veneered zirconia ceramic fixed partial dentures. J Esthetic Restor Dentistry 32(4):395–402. 10.1111/jerd.12568 10.1111/jerd.12568 31999068

[24] Hensel F, Koenig A, Doerfler HM, Fuchs F, Rosentritt M, Hahnel S (2021) CAD/CAM resin-based composites for use in long-term temporary fixed dental prostheses. Polymers 13(20). 10.3390/polym13203469 10.3390/polym13203469 PMC8539791 34685228

[25] Park MG (2023) Effect of low-temperature degradation treatment on hardness, color, and translucency of single layers of multilayered zirconia. J Prosthet Dent. 10.1016/j.prosdent.2023.01.023 10.1016/j.prosdent.2023.01.023 36804392

[26] Murbay S, Yeung SKW, Yip CY, Pow EHN (2023) Assessing enamel wear of monolithic ceramics with Micro-CT and intra-oral scanner. Int Dent J 73(4):496–502. 10.1016/j.identj.2022.10.007 36428104 10.1016/j.identj.2022.10.007 PMC10350602

[27] Kim YJ, Ko KH, Park CJ, Cho LR, Huh YH (2022) Connector design effects on the in vitro fracture resistance of 3-unit monolithic prostheses produced from 4 CAD-CAM materials. J Prosthet Dent 128(6):1319e1311–1319e1310. 10.1016/j.prosdent.2022.09.018 10.1016/j.prosdent.2022.09.018 36334990

